# The Responsibility of Physician to Patient, Considered from a Legal Point of View

**Published:** 1893-05

**Authors:** William L. Marcy

**Affiliations:** Buffalo, N. Y.


### THE RESPONSIBILITY OF PHYSICIAN TO PATIENT, CONSIDERED FROM A LEGAL POINT OF VIEW.

By WILLIAM L. MARCY, Esq., Buffalo, N. Y.

The learned professions have nothing to gain by surrounding their practice with mysteries; the legal profession has been slowly discarding old wigs and gowns, forgotten words, and obsolete practices; no lawyer could ever tell why the penal clause in every bond you sign with a mortgage on your house is made in double the sum that is agreed to be paid. You will not regard this allusion as personal, for in this real estate age every man has a lot and every lot has its mortgage. What is the reason for this penal clause? It has only a poor feminine excuse—it is so, forsooth, because it is so. After years of uselessly treading this path our fathers trod, some legislator with a thought under his cap will write a law on the statute books burying this foolishness and marking on its tombstone "*obsolete.*" Yet, all professions are slow to change and jealous of innovations. The elders want not to unlearn and forget, to acquire new habits of thought and speech, and so new truth makes slowly. But change we must, and he who is past learning a better way is surely past the meridian of his usefulness. The attorney does not fear the simplification of practice and procedure, the weeding out of useless words and phrases. That handy volume, "Every Man His Own Lawyer," has evolved an adage which works out a solution, for it has also found a fool for a client. And is it not pardonable if I ask of this very intelligent body of men, what good end is gained by deluding your patients with bread pills and *aqua pura.* Would not a cheery word of encouragement, a vigorous pat on the back or chuck under the chin serve quite as well? Poor humanity is so credulous, it loves so to be humbugged, yet I like my doctor to be truthful.

Law is an evolution; each discovery injects a new problem into the social organism. With the introduction of railroads, for instance, a new factor came upon the scene of action; from that day until the present, thousands of cases have been tried in the courts, presenting an infinite variety of situations and questions that have necessarily arisen in the development of that great industry. New rules had to be made and applied; partly in the legislature and partly in the courts this branch of the law has grown into copious proportions. It is not always perfect or har-

1. Read before the Section on Surgery of the Buffalo Academy of Medicine, April 4, 1893.

monious, but, on the whole, it has developed and will develop into a just and wise system. In the main, law is impressed upon an industry by those who are not engaged in its pursuit; it is an intelligent effort to follow in the line of justice and equity, and do that which common sense and common prudence say are right and reasonable. Truth and justice must be in every law. When the history of our day and generation is written, it will be mainly from our laws and their enforcement that this civilization will be judged. Candidly, do you think our children's children will look down upon us and wonder at some of our Egyptian practices?

Now, to talk to my text, I have been requested, within the limit of fifteen minutes, to make clear and plain what measure of justice has been doled out to the doctors, what rule of responsibility your patients, in court and legislature, have imposed. The legal principles involved have no exclusive application to the department of surgery, but it doubtless happens that in the other departments of medicine the results of the physician's error are not so readily discoverable, and, hence, are not often the subject of litigation.

The patient gets well, and he is glad enough to get out, or, if he dies, the tombstone covers, at the same time, occasion, cause, and effect.

With surgery, however, the patient can look at the mutilated member. There is a certain community of misery and suffering which brings the good neighbor in to visit and to gossip, and which prompts the kindly offer of home-made remedies. It leads inevitably to a critical analysis of the case in particular, and a comparison with all others known, and soon the patient is told how Dr. Jones performed the same operation successfully on Mrs. Smith. When your patient recovers, and finds that his arm is stiff and he cannot use it, or that the injured leg is two inches shorter than its brother, he inquires into the situation, and his inquiries lead, occasionally, to the lawyer's office. How will he be advised there? That is the question. The first inquiry is, Did the attending physician possess that reasonable degree of learning and skill which is ordinarily possessed by men engaged in the practice of that profession? This query is, generally, easy of answer. In cities, where the tendency in the profession is into specialties, the general practitioner rarely treats surgical cases, and the reputation of the doctor among his fellows is readily ascertainable. The law requires him to possess, not the highest, not the best qualifications but ordinary professional skill. The reason for this is obvious. No question depending upon mere opinion can be exact, and no given number of men would agree as to what constitutes perfect skill. A diploma is almost *prima facie* evidence of competency, so that the measure of responsibility, in this respect, is liberal to the extreme. But in the country, where the physician is called upon to treat diseases and disorders in both physics and surgery, the comparison of skill with ordinary skill in the profession may involve more hazard to the doctor. The shrewd attorney will inevitably make this comparison with the skilled, practiced specialist. There is an element of unfairness in this. While our courts have not definitely held that such a comparison of skill must be made with the ordinary skill which a particular locality offers, when a case is presented where the doctor is called upon to act in an emergency without opportunity being given to call in consultation a more experienced practitioner, the measure of responsibility will, doubtless, be modified to meet such a condition. But where there is ample time for deliberation and preparation, then the number of similar surgical cases treated by the country physician, his knowledge and application of improved methods, may enter into and make the question of his skill one of fact for the determination of a jury. In a case cited in the law reports, the doctor was led, on his cross-examination, to say that he did not consider himself an expert in surgery. If this take the form of an admission, or be fairly deducible as an inference from his want of opportunity to treat similar cases, and a failure to apply and use the more modern treatment and approved methods, then the question may become one that must be left for the decision of a jury.

The next question is, Did the attending physician exercise his best judgment and use reasonable and ordinary care and diligence in the exercise of his skill? A bad result is not sufficient of itself to create liability. A physician or surgeon does not guarantee the result of any treatment. He undertakes to possess ordinary professional skill, to exercise this skill with reasonable diligence and care and the use of his best judgment, and this being done, his whole duty is discharged. A bad result may be some evidence of improper care or faulty treatment, but proof must be given of the lack of ordinary professional skill, or a failure to observe reasonable care and diligence or the exercise of sound judgment producing this result.

As stated by our courts the rule is:

A physician assumes to have, and is required to possess, at least, ordinary professional intelligence and skill, and with his best judgment to exercise it in the treatment of a patient. He is not required to insure results or to guarantee that the consequences will be beneficial. While the responsibility of the medical practitioner and surgeon is great, and care proportionally should be observed in the exercise of his professional employment, when his errors are those of judgment only, if he keeps within recognized and approved methods he is not liable for their consequences.

Mere error in judgment, as disclosed by consequences, produces no liability when there is no departure from a professionally approved line of action. By this is not meant that every skilled member of the profession would or should necessarily adopt precisely the same remedy for a like condition. Such an arbitrary rule would have the effect to unduly qualify the right of exercising judgment required by attending circumstances, temperament, and physical condition of patients, and the complication of diseases and physical troubles involved, but in the main, he should observe and take the benefit of the past experience and learning of the profession and adopt them as the rule of action when practicable rather than new and experimental methods. Much must necessarily rest in the judgment of the attending physician.

The difference of opinion in respect to the propriety of a specific treatment may be one of judgment rather than skill, and, therefore, insufficient to present the question of liability upon the charge of malpractice as one of fact, else it would be difficult to find a cause of unfavorable results from medical treatment which would not furnish a question in that respect for the jury.

To establish negligence, as a rule, there must be a comparison of the diagnosis, history and result of the case in controversy with other similar cases reported, and with the common experience of others learned in the profession and recognized in professional literature. There may be some conspicuous act of carelessness, like a bandage or splint improperly applied; but the doctor is, after all, the judge of his fellow-practitioner—in the capacity of an expert witness, he gives an opinion as to the commouly accepted and accredited methods of treatment and his judgment of the expected result. And where trustworthy opinion is advanced that there is some neglect or fault, the question ceases to be one of law and becomes a controverted question of fact, which must be submitted to a jury.

I have been frequently asked why, if these cardinal principles are shown to have been sedulously observed by the doctor, his case is not decided by the judge, as a matter of law, and not submitted to the uncertainties of the jury. If this question comprehended all in the case, that would be the inevitable result. The patient and plaintiff must show an infraction of these well-settled principles, in order to establish any liability, and it is only where the result produced is shown by some evidence to have been occasioned either by lack of skill or want of proper care and judgment that the issue becomes a question of controverted fact and renders liability possible.

It is also to be observed that the same rules and principles apply when the service is gratuitous, and the physician who has undertaken a case may not leave his patient without giving full and ample opportunity to call in some other when he retires.

A novel situation may arise in the future when learned Christian scientists may attempt to justify their treatment of a patient by resorting to the rules of ordinary professional skill; he will have to search diligently outside of the Scriptures for the faith that lies within him. Professional courtesy has shielded many a bungler, but we are all conscious that no man has risen to eminence in the medical profession without years of service and probation, in which there not only may have been, but undoubtedly were, many errors and mistakes; and in remedying the errors and mistakes of the past, the physician has grown to the full measure of his power. It is only by a tolerant, a liberal, a beneficent policy that the medical profession has given to the world its Gross and its Hamilton, and in your midst has produced a Park and a Mynter.

